# Supplementary material for: Cohesins and condensins orchestrate the 4D dynamics of yeast chromosomes during the cell cycle
Source: EMBO J. 2017 Jul 20;36(18):2684–97. doi: 10.15252/embj.201797342 (PMC5599795; doi:10.15252/embj.201797342)
Supplement: Supplementary file 1 — Appendix [file EMBJ-36-2684-s001.pdf]

Appendix

Table of content

Appendix Figure S1	Page 2
Appendix Figure S2	Page 3
Appendix Figure S3	Page 4
Appendix Figure S4	Page 5
Appendix Supplementary Methods	Page 6

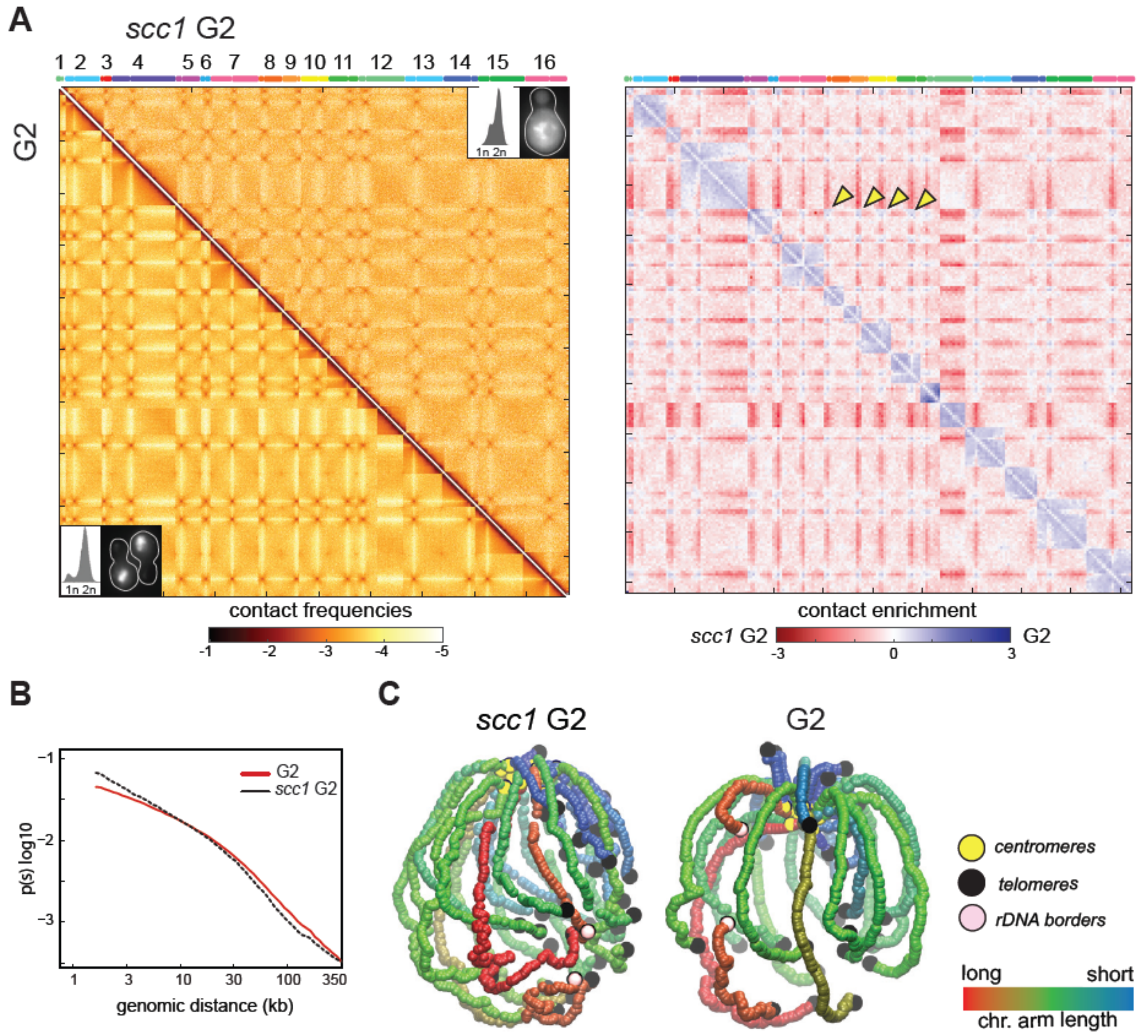

Appendix Figure S1. Cohesin required for genome-wide chromatin organization during replication.

(**A**) Left panel: Hi-C contact maps of cell populations G1 released and processed in G2 wild-type (G2, bottom left) and in cohesin depleted (*scc1* G2, upper right) cells. The corresponding FACS profiles as well as representative DAPI-stained cells are displayed in boxes on the bottom left and upper right corners, respectively. X and y axis represent the 16 chromosomes of the yeast genome. Same color code as in Figure EV1. Right panel: log-ratio of G2 and *scc1* G2 contact maps. Yellow arrowheads: inter-centromere contacts. The color code reflects the enrichment in contacts in one population with respect to the other. (**B**) Contact probability p(*s*) decay in G2 and *scc1* G2. (**C**) 3D average representations of the Hi-C contact maps of panel (**A**). Color code represents chromosomal arm length and centromeres, telomeres and rDNA flanking regions are highlighted.

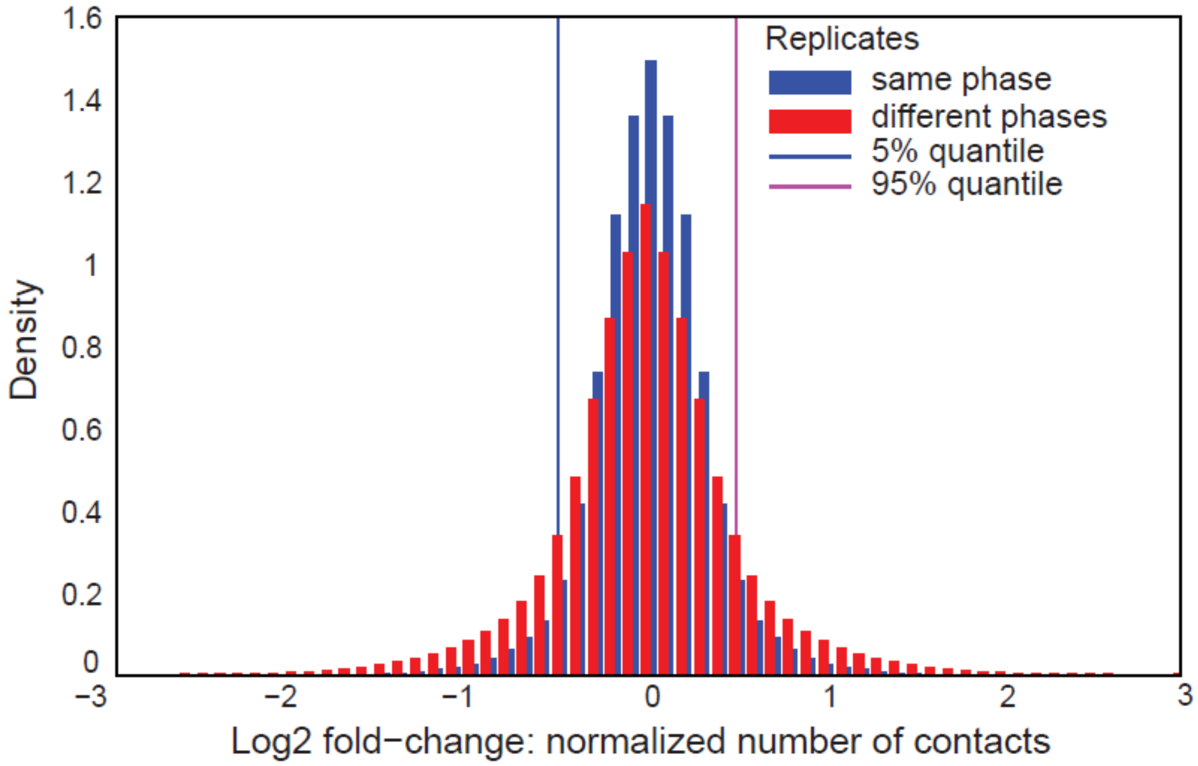

Appendix Figure S2. Analysis of the variability of Hi-C data.

Distribution of the fold-change in contacts made by bins in over different conditions. Blue: fold change in contacts in replicates. Red: fold change in contacts between all non-replicated conditions. This results backs the co-localization in the PC space of replicates in the PCA.

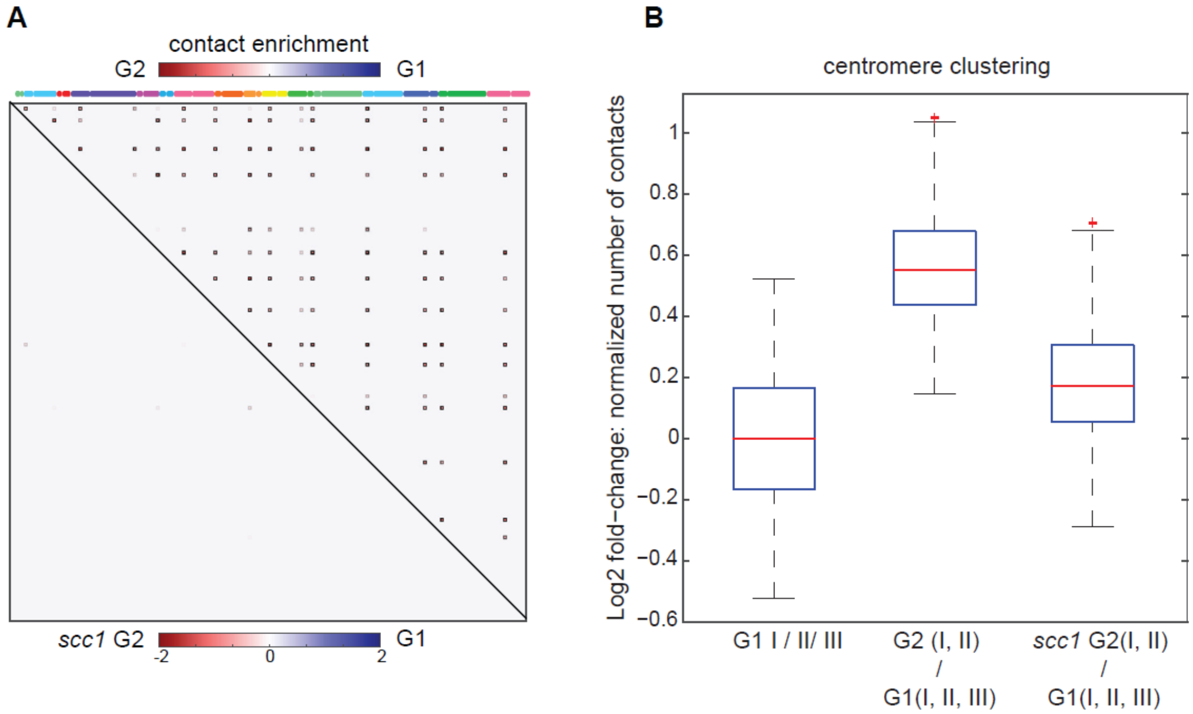

Appendix Figure S3. Centromere clustering is cohesin-dependent.

A) Positions within the contact map of bins whose specific variations in contacts in trans will be investigated between two conditions (here, the 16 bins corresponding to centromeres, between G1 and G2 [top right] and between *scc1* and G1 [bottom left]). **B**) Boxplots representing the variation in number of normalized contacts for a subset of bins between different contact maps (corresponding to different conditions). Left: variations between three G1 replicates. Middle: variations between G2 and G1 cells replicates. The relative Wilcoxon test provides a p-value $< 10^{-10}$, supporting an increase in contacts in G2 compared to G1. Right: variations between *ssc1* arrested cells and G1 replicates.

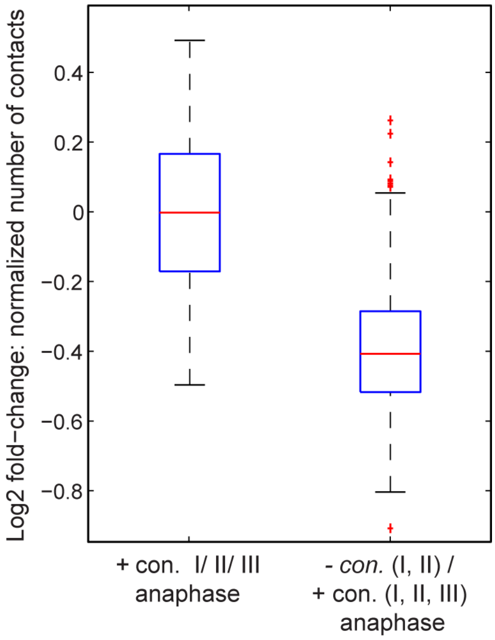

Appendix Figure S4. **Condensins increase centromere clustering in anaphase.**

Left boxplot shows the centromere contact variability between anaphase replicates (*cdc15* I, II, III) in presence of condensins (+con.). Right boxplot shows the variation of contacts between centromeres between *cdc15* replicates and condensin defective strains arrested in anaphase (*cdc14* and *smc2 cdc15*; -con. I, II). The boxplots show that the centromere cluster in anaphase is condensin-dependent ($P < 0.05$).

Appendix Supplementary Methods

Generation of Hi-C libraries. Aliquots of 1-3 x 10^9 cells in 150 ml YPD/synthetic medium were fixed in 3% formaldehyde (Sigma) for 20 min at room temperature and quenched with 25 ml glycine 2.5 M for 20 min at 4°C. Cross-linked cells were recovered through centrifugation, washed with YPD and a 150 mg pellet was stored at -80°C. The pellet was thawed on ice and incubated for 30 min in 10 ml of sorbitol 1M, DTT 5mM and Zymolyase 100T (C_{Final}=1 mg/ml; Armsbio). Spheroplasts were washed once with 5 ml sorbitol 1M, once with 5 ml 1X RE buffer (DpnII NEB buffer) and finally suspended in 3.5 ml of 1X RE buffer. The spheroplasts were treated with 3% SDS for 20 min at 65°C and the lysate was digested overnight with DpnII (C_{Final}=450 U/pellet; NEB) at 37°C. The digestion was centrifuged for 20 min at 18000 g, the supernatant discarded and the pellet suspended in 400 µL of water. The 5' overhangs from DpnII digestion were filled in using dNTP 30 µM (biotin-14-dCTP, dATP, dGTP and dTTP; Invitrogen), at 37°C for 45 min. The biotinylated DNA fragments were ligated by T4 DNA ligase (C_{Final}=250 Weiss U/pellet; Thermo Scientific) for 4 h at 16°C. DNA purification was achieved through an overnight incubation at 65°C in presence of proteinase K 250 µg/ml and EDTA 6.2 mM, followed by a phenol/chloroform extraction on the precipitated DNA and an RNAse A DNase-free 500 µg/ml treatment. The biotinylated but not ligated DNA fragments were removed by T4 DNA polymerase (C_{Final}=5 U/pellet; NEB) treatment. Hi-C DNA libraries were 500 bp sheared, using CovarisS220 apparatus, and the biotin-labeled fragments were selectively captured by Dynabeads Myone Streptavidin C1 (Invitrogen). The resulting libraries were used as template for the llumina amplification by PE-PCR primers and paired-end sequenced on the NextSeq500 or HiSeq 2000 Illumina platforms (2x75 or 2x150 bp kits; see Table EV2).

Elutriation (recovery of G1 cells). 800 ml overnight culture was centrifuged, washed in 1X PBS and pelleted cells were suspended in 1000 ml of fresh YPD for 2 h at 30°C. This additional growing step allowed cells in stationary phase to reenter exponential phase before being elutriated. For each elutriation experiment, 1.2-1.8 x 10^{11} cells were washed and suspended in 30 ml of 1X PBS and injected in the 40 ml elutriation chamber at an average flow rate ranging from 20 ml/min to 25 ml/min (MasterFlex L/S pump from Cole-Parmer), at 2,500 r.p.m. and 23°C. Cells were then left to equilibrate in 1X PBS for 45 min at a constant

flow and rotational speed. To start collecting the first fractions containing the small G1 cells, a periodic 2 ml/min increment of the flux was applied between each fraction. The resulting 600 ml fractions were centrifuged and approximately 2.5 x 10^9 G1 cells/fraction were recovered. Before fixating the G1 state, cells were suspended in fresh YPD at 30°C for 30 min, so they could recover from their stay in PBS during the elutriation. To minimize the potential variability introduced by the age heterogeneity of the bulk population, G1 daughter cells were used as starting point for all cell cycle synchrony and in combination with genetic and chemical synchronization methods (see below).

Synchronization through thermosensitive mutations. Synchronizations using thermosensitive (ts) *cdc* strains (Hartwell *et al*, 1973) were all performed starting from elutriated G1 daughter cells growing in non-permissive temperature conditions designed to arrest the progression of the cycle at specific phases.

The G1/S checkpoint (*cdc6-1* mutation; YKL054 strain) was activated by growing cells overnight at 25°C, restarted in fresh YPD media and elutriated while in exponentially growing stage, still at 25°C. The elutriated G1 cells were incubated in fresh YPD at the non-permissive temperature of 37°C for 3 h. To study non-replicated mitotic chromosomes, the *cdc6-1* arrested cells were maintained in non-permissive growing conditions for an extended period of 6 h. During this time period, G1 cells bypass the G1/S checkpoint and proceed into M phase without having replicated their chromosomes.

A similar protocol was applied to *cdc14-3* (YKL052) and *cdc15-2* (YKL053) G1 cells exposed to non-permissive temperature of 30°C and 37°C after elutriation, respectively. *Cdc15* and *cdc14* arrested cells blocked into anaphase were shifted at the permissive temperatures of 25°C and 23°C, with different time-points were sampled after release (YKL052: 30 min; YKL053: 20 min, 40 min and 60 min). The synchrony of each time point (in G1/S, anaphase and release) was monitored with flow cytometry and microscopy.
