# Supplementary material for: Cohesins and condensins orchestrate the 4D dynamics of yeast chromosomes during the cell cycle
Source: EMBO J. 2017 Jul 20;36(18):2684–97. doi: 10.15252/embj.201797342 (PMC5599795; doi:10.15252/embj.201797342)
Supplement: Supplementary file 2 — Expanded View Figures PDF [file EMBJ-36-2684-s002.pdf]

Expanded View Figures

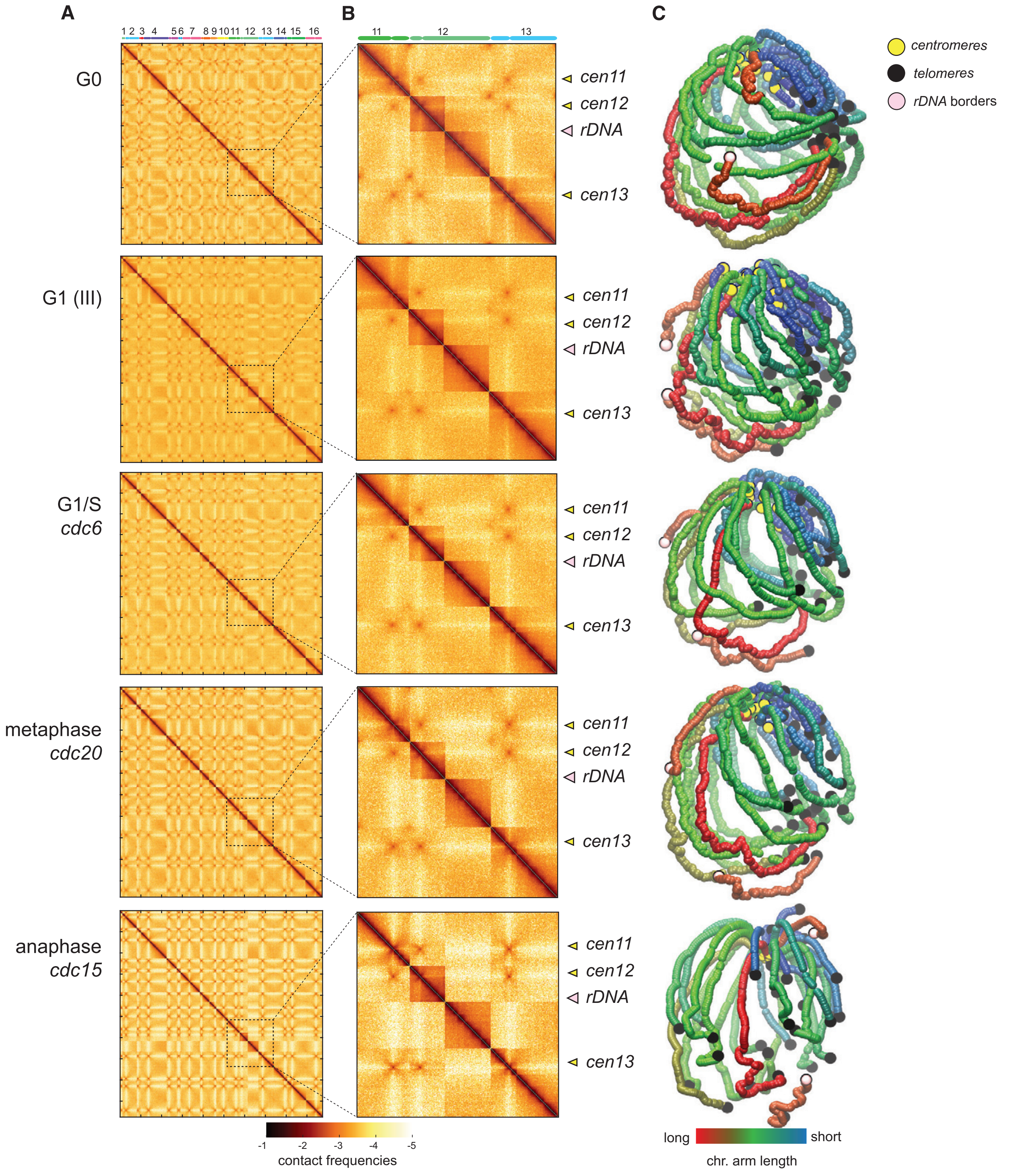

Figure EV1.

Figure EV1. Contact maps and 3D genome representations of the five cell cycle synchronization states.

A, B Contact maps generated from synchronized cell populations described in this study, with each vector (or bin) corresponding to 5 kb. *x*- and *y*-axis represent the 16 chromosomes of the yeast genome, displayed atop the maps. Brown to yellow color scales reflect high to low contact frequencies, respectively (log10). Magnification panels in (B) show variations of the contact frequencies between synchronized populations. Yellow and pink arrowheads point at centromeres and rDNA positions, respectively.

C 3D average representations of the Hi-C contact maps of synchronized cell populations of panel (A). The color code represents the chromosomal arm length, and centromeres, telomeres, and rDNA flanking regions are highlighted.

Figure EV2. Contact maps and 3D genome representations during replication.

A, B Contact maps recovered from cell populations undergoing replication after G1 release. For each contact map, the FACS profile is displayed. *x*- and *y*-axis represent the 16 chromosomes of the yeast genome. The same color code as in Fig EV1. Magnification panels in (B) highlight changes of the contact frequencies during S-phase progression. Yellow and pink arrowheads point at centromeres and rDNA positions, respectively.

C 3D average representations of the Hi-C contact maps of synchronized cell populations of panel (A). The color code represents the chromosomal arm length, and centromeres, telomeres, and rDNA flanking regions are highlighted.

© 2017 The Authors

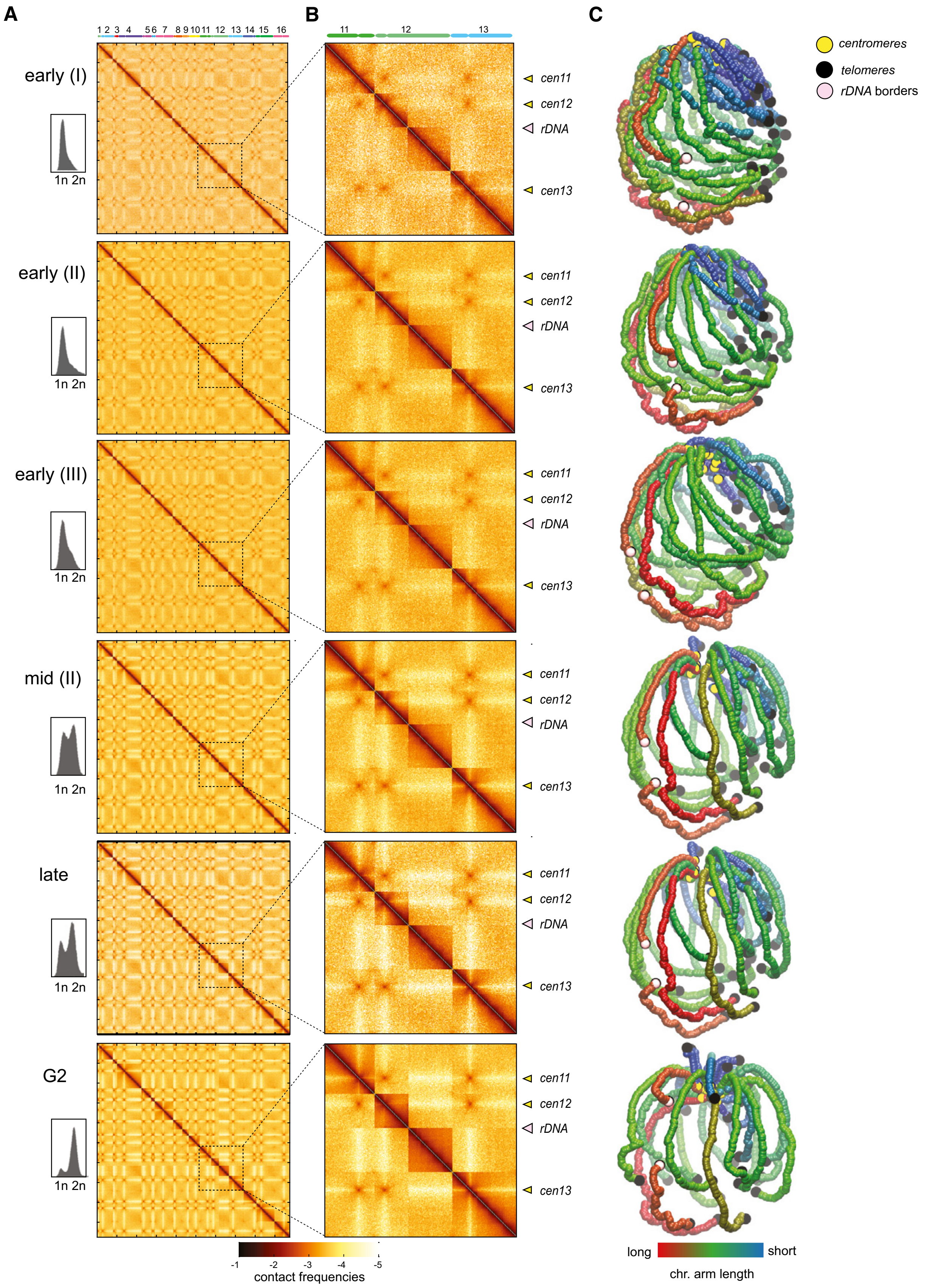
A
B
C
1 2 3 4 5 6 7 8 9 10 11 12 13 14 15 16
11
12
13
early (I)
early (II)
early (III)
mid (II)
late
G2
1n 2n
cen11
cen12
rDNA
cen13
centromeres
telomeres
rDNA borders
-1
-2
-3
-4
-5
contact frequencies
long
short
chr. arm length

Figure EV2.

Figure EV3. Contact maps and 3D genome representations during M phase.

A, B Contact maps of cell populations synchronized in metaphase (*cdc20*) and anaphase (*cdc15*) and released into mitosis from *cdc15* block (+20 min, +40 min, and +60 min). The corresponding FACS profiles and representative DAPI-stained cells are displayed on the left on the maps. x- and *y*-axis represent the 16 chromosomes of the yeast genome. The same color code as in Fig EV1. Magnification panels in (B) display variations of the contact frequencies during mitotic progression. Yellow and pink arrowheads point at centromeres and rDNA positions, respectively.

C 3D average representations of the Hi-C contact maps of panel (A). The color code represents the chromosomal arm length, and centromeres, telomeres, and rDNA flanking regions are highlighted.

 © 2017 The Authors

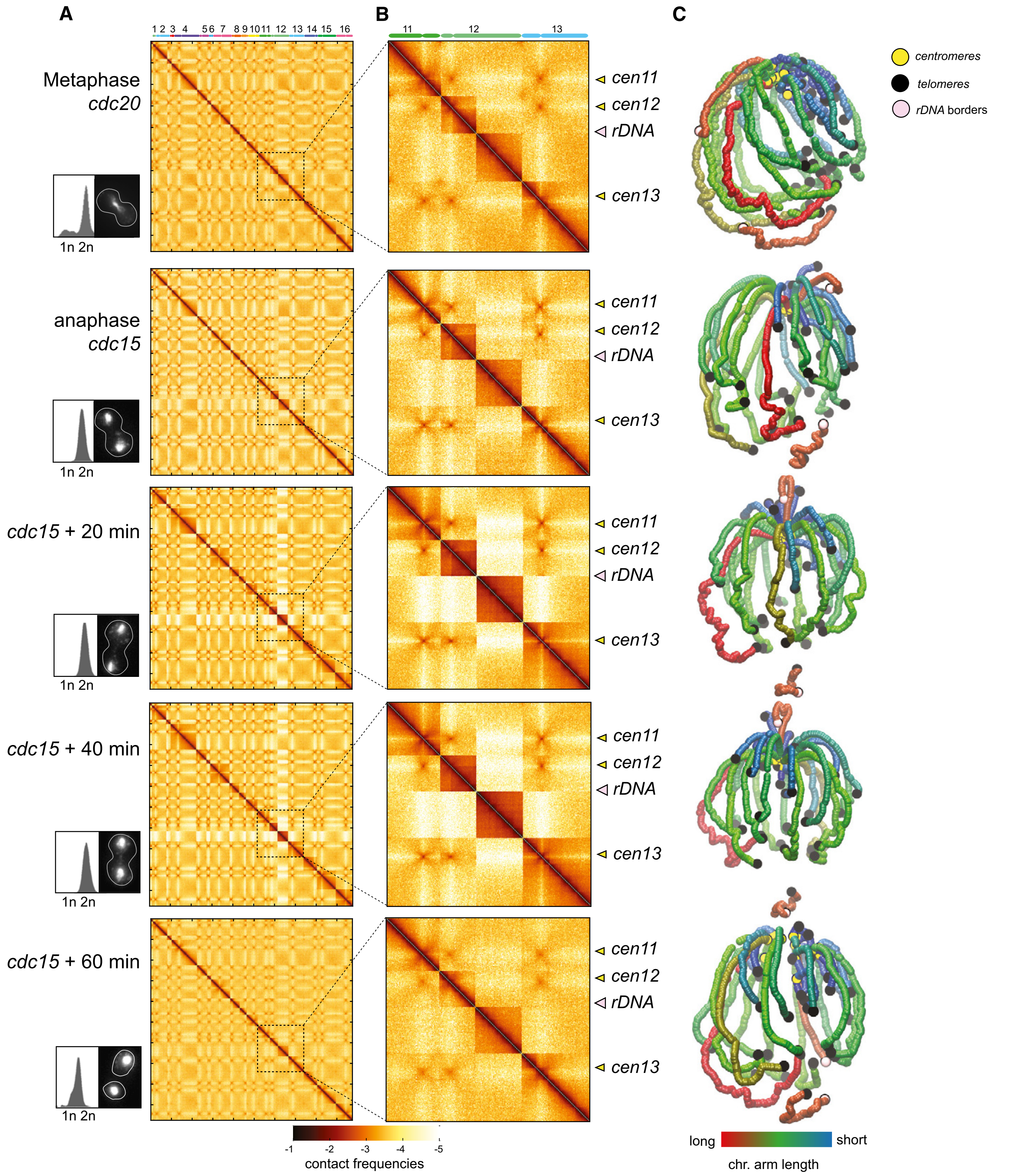
A
B
C
1 2 3 4 5 6 7 8 9 10 11 12 13 14 15 16
11 12 13
Metaphase cdc20
anaphase cdc15
cdc15 + 20 min
cdc15 + 40 min
cdc15 + 60 min
1n 2n
cen11
cen12
rDNA
cen13
centromeres
telomeres
rDNA borders
-1 -2 -3 -4 -5
contact frequencies
long
short
chr. arm length

Figure EV3.

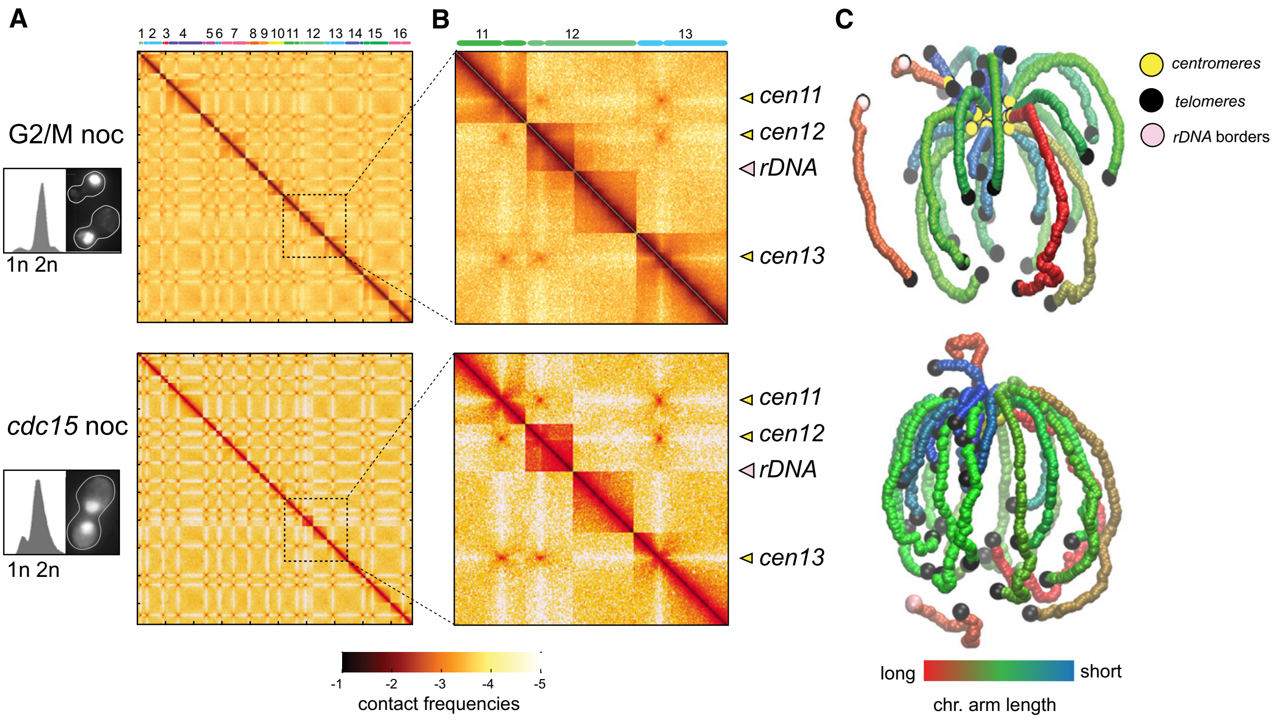

Figure EV4. Nocodazole affects chromosome 12 conformation.

A, B Contact maps of G1 synchronized cell populations released either in the presence of nocodazole (G2/M noc) or at *cdc15* non-permissive temperature followed by a nocodazole treatment (*cdc15* noc). The corresponding FACS profiles and representative DAPI-stained cells are displayed on the left on the maps. x- and *y*-axis represent the 16 chromosomes of the yeast genome. The same color code as in Fig EV1. Magnification panels in (B) display variations of the contact frequencies. Yellow and pink arrowheads point at centromeres and rDNA positions, respectively.

C 3D average representations of the Hi-C contact maps of panel (A). The color code represents the chromosomal arm length, and centromeres, telomeres, and rDNA flanking regions are highlighted.

 © 2017 The Authors

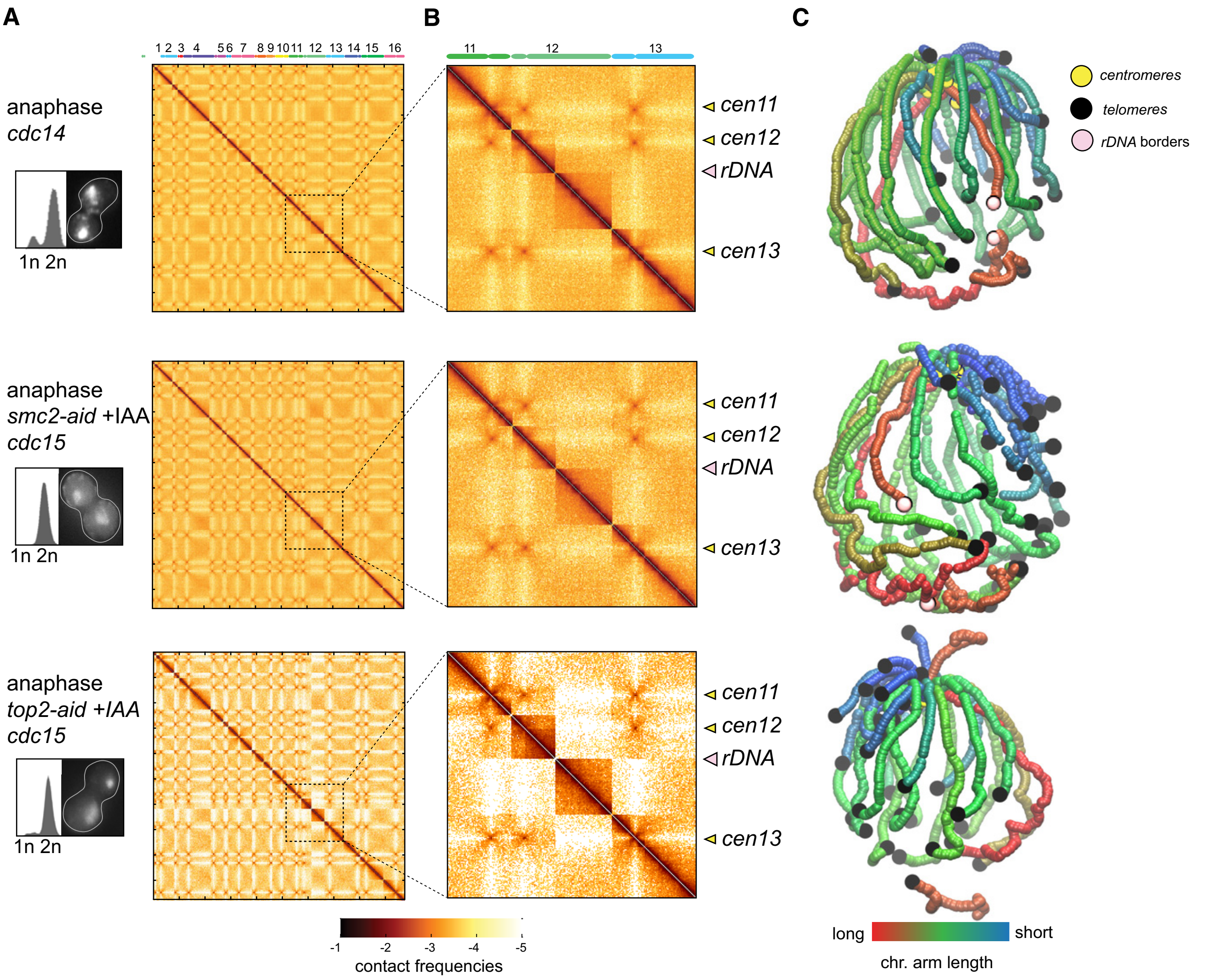

Figure EV5. Condensin and decatenation influence on chromatin structure during mitosis.

A, B Contact maps of cell populations arrested in anaphase, either defective with condensation (*cdc14* or *smc2-aid cdc15* +IAA) or depleted of topoisomerase 2 (*top2-aid cdc15* +IAA). The corresponding FACS profiles and representative DAPI-stained cells are displayed on the left on the maps. x- and *y*-axis represent the 16 chromosomes of the yeast genome. The same color code as in Fig EV1. Magnification panels in (B) display variations of the contact frequencies. Yellow and pink arrowheads point at centromeres and rDNA positions, respectively.

C 3D average representations of the Hi-C contact maps of panel (A). The color code represents the chromosomal arm length, and centromeres, telomeres, and rDNA flanking regions are highlighted.

© 2017 The Authors
